# Hypothesis: Sam68 and Pygo2 mediate cell type-specific effects of the modulation of CBP-Wnt and p300-Wnt activities in Colorectal Cancer Cells

**DOI:** 10.7150/jca.59726

**Published:** 2021-06-16

**Authors:** Michael Bordonaro

**Affiliations:** Department of Medical Education, Geisinger Commonwealth School of Medicine, 525 Pine Street, Scranton, PA 18509, USA.

**Keywords:** Sam68, CBP, p300, ICG-001, colorectal cancer, Wnt signaling, butyrate

## Abstract

The preventive activity of dietary fiber against colorectal cancer (CRC) may be in part mediated by the fermentation product of fiber, butyrate, a histone deacetylase inhibitor (HDACi) that induces CRC cell growth arrest and apoptosis. This action of butyrate, and other HDACis, is in part due to the hyperactivation of the deregulated Wnt activity found in the relevant CRC cell lines. The histone acetylases CBP and p300 interact with beta-catenin; and the relative levels of CBP-Wnt vs. p300-Wnt activity influences CRC cell physiology. It has previously been observed that there are cell type-specific differences in how cotreatment with butyrate and ICG-001, an agent that blocks CBP-Wnt activity allowing for p300-Wnt activity, affects CRC cell physiology. These differences may have clinical significance in dealing with treatment of CRC patients with ICG-001-like agents. Sam68 is a factor differentially expressed in cancer cells, with higher expression in cancer cell lines that have cancer stem cell (CSC)-like properties. Sam68 expression sensitizes cancer cells to ICG-001 treatment, as ICG-001 enhances nuclear localization of Sam68, where binding between Sam68 and CBP diminishes CBP-beta-catenin binding and thus CBP-Wnt activity. Pygo2 is a chromatin effector involved with Wnt signaling that is differentially acetylated by CBP and p300; thus CBP-mediated acetylation localized Pygo2 to the nucleus where it functions in transcriptional activation, while p300-mediated acetylation localizes Pygo2 to the cytoplasm. This paper proposes the hypothesis that Sam68 and Pygo2 are responsible for cell type-specific response of CRC cell lines cotreated with ICG-001 and butyrate as well as other HDACis. Further, experiments are proposed to evaluate this hypothesis and consider possible expected results that could be obtained from such studies.

## Introduction

### Fiber, butyrate, Wnt signaling, and butyrate resistance

A higher daily intake of dietary fiber reduces colorectal cancer (CRC) risk; butyrate, a histone deacetylase inhibitor (HDACi) that is a breakdown product of fiber 1-11, is thought to at least partially mediate this protective activity. CRC physiology is significantly affected by *in vitro* treatment with HDACis, including butyrate, which typically induces cell cycle arrest, differentiation, and/or apoptosis 1,2,12-17. Deregulated Wnt signaling, most typically occurring as a result of mutations in the *APC* or *beta-catenin* genes, initiates most cases of CRC 18-28. Given that excessive activation of oncogenic signaling can promote apoptosis 29, it is not surprising that Wnt activity displays a continuum of phenotypic effects (“just right hypothesis”) in which colonic neoplasia is promoted by moderate Wnt signaling levels 30, and various studies have shown that excessive levels of Wnt activity result in apoptosis 17, 31-36. Importantly, *in vitro*, butyrate and other HDACis hyperactivate Wnt signaling in CRC cells, which is causally associated with enhanced apoptosis and suppressed clonal growth and cell proliferation 1,2,17,37. Of course, CRC can still develop despite a high-fiber diet 6-8, suggesting that resistance to butyrate frequently develops during the process of colonic tumorigenesis. To evaluate this possibility, a HCT-116 CRC cell line was made resistant to 5 mM butyrate, which is a physiologically relevant concentration well within the levels found in the colonic lumen, and which efficiently induces Wnt hyperactivation, apoptosis, and cell growth arrest *in vitro*. These cells, designated as “HCT-R,” exhibit suppressed levels of Wnt activation, apoptosis, and growth arrest after exposure to butyrate and are also cross-resistant to other, clinically relevant, HDACis 2. These cells also exhibit upregulation of Tcf3, which represses Wnt activity, and they are also deficient in expression of p300 39.

### CBP, p300, Sam68, and Pygo2

ICG-001-like agents, such as ICG-001 and its analog CWP, block CBP-Wnt signaling by inhibiting the association between CBP and beta-catenin 40-42. Sam68 is a binding partner for CBP and represses expression of the Wnt target gene cyclin D1 41,42, and is overexpressed in breast and colon cancer cell lines that have cancer stem cell (CSC) properties, but not in non-CSC lines (e.g., Sam68 expression is higher in the CSC-like HT29 CRC cell line compared to the non-CSC-like SW480 line) 42. Those CSC-like lines are more sensitive to the cell growth-inhibiting effects of ICG-001 and CWP and exogenous expression of Sam68 in the non-CSC-like MCF-44 breast cancer cell line sensitizes those cells to CWP 42. Comparing normal (PSC) and neoplastic (t-PSC) human pluripotent stem cells, levels of CBP and Sam68 are approximately similar, but t-PSCs exhibit much greater sensitivity to ICG-001 and CWP compared to PSCs. Those agents cause increased nuclear localization of Sam68 specifically in the t-PScs, resulting in increased association of CBP with Sam68, decreased association of CBP with beta-catenin, and thus decreased CBP-mediated histone acetylation 42. The ability of ICG-001-like agents to decrease occupancy of CBP at Wnt target gene promoters most likely results from the ability of those agents to enhance nuclear localization of Sam68, where it interferes with CBP-Wnt activity 42. Pygopus 2 (Pygo2) is a chromatin-modulating factor that plays a pivotal role in Wnt transcriptional complexes 43; further, Pygo2 interacts with CBP and p300 and is acetylated by those factors, a modification that affects levels of Wnt activity 43. Acetylation of Pygo2 by CBP or p300 has different effects; CBP-induced acetylation promotes nuclear localization of Pygo2, while p300-induced acetylation localizes Pygo2 to the cytoplasm 43. Given that the activity of Pygo2 is in the nucleus, this variable response may explain why CBP-Wnt signaling promotes cell proliferation, while p300-Wnt signaling promotes differentiation 40,43.

The joint effects of Sam68 and Pygo2 may influence the cell type-specific effects of ICG-001 observed in different CRC cell lines 44-47. Thus, we have observed cell type-specific effects (e.g., apoptosis) of ICG-001 in CRC cell lines, particularly in conjunction with cotreatment with butyrate 44-47. Thus, in SW620 CRC cells (derived from a metastasis of the non-CSC SW480 line tumor), ICG-001 interferes with butyrate-induced apoptosis, while this effect is not observed in HCT-116 CRC cells that contain a CSC-like subpopulation 49. One possibility is that these cell type-specific differences are in part mediated by Sam68.

Sam68 and Pygo2 may mediate effects of CBP-Wnt and p300-Wnt signaling on resistance to butyrate (and other HDACis). HCT-116 cells made resistant to butyrate by continuous exposure to that agent (HCT-R cells) exhibit repressed expression of p300 45 and we have demonstrated that the HCT-116 p300 KO lines D10 and F5 48, particularly F5, exhibit butyrate resistance 47. If loss of p300 induces significant butyrate resistance, then restoration of p300 expression in a knockout line would be expected to enhance butyrate sensitivity. Butyrate sensitivity was therefore evaluated in a “rescue” line generated by stable expression of p300 48 in the F5 knockout line 47. Rescue cells exhibited a restoration of fold-upregulation of Wnt activity by butyrate compared to the F5 knockout line. Rescue cells exhibited lower-fold induction of apoptosis compared to the HCT-116 line; however, when comparing the final levels of caspase activity in the presence of butyrate, Rescue cells exhibit the highest levels of butyrate-mediated apoptosis, higher than both HCT-116 cells and the knockout line. Thus, restoration of p300 expression resulted in a marked increase in the final level of apoptosis.

In addition, the ability of butyrate to repress cell proliferation was enhanced by rescue expression of p300. Proliferation in the presence of butyrate was similar between HCT-116 and Rescue cells, while, as expected, F5 knockout cells exhibited greater proliferation after butyrate treatment than HCT-116 or Rescue cells. The three cell lines were also compared with respect to clonogenic growth, which is influenced by both proliferation and apoptosis 47. Treatment with butyrate resulted in a 55% reduction in colony formation in HCT-116 cells, while F5 cells exhibited a 36% reduction in clonal growth. Rescue cells were more sensitive to butyrate, exhibiting a 63% reduction in clonal growth. These findings suggest that p300-mediated Wnt signaling is required for optimal hyperactivation of Wnt signaling and repression of cell proliferation by butyrate.

Thus, p300 deficiency associated with butyrate resistance 45,47,48 may favor CBP-induced acetylation of Pygo2 and, hence, nuclear localization leading to differential gene expression. Therefore, Pygo2 may be an important mediator of the differential effects of CBP and p300 in CRC, and may play a crucial role in butyrate resistance involving p300 deficiency.

### Hypothesis

**Figure 1:**
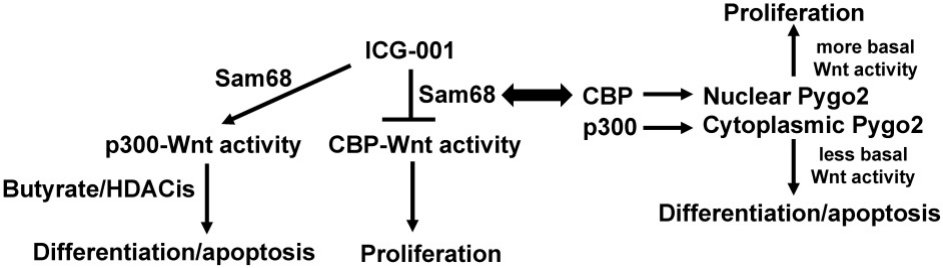
** Diagram outlining the fundamental hypothesis concerning interactions between Sam68, Pygo2, and Wnt signaling.** CBP-mediated Wnt signaling, which is inhibited by ICG-001, promotes colonic cell proliferation, while p300-mediated Wnt signaling, which can be potentiated by the suppression of CBP-Wnt activity, tends to promote colonic cell proliferation and apoptosis. Apoptosis of colorectal cancer cells that is induced by butyrate (and likely other HDACis) is at least partially dependent upon intact p300 activity, as we have observed an association between butyrate resistance and repressed expression of p300. Sam68 mediates the suppression of CBP-Wnt activity by ICG-001 through Sam68-CBP association, likely promoting p300-Wnt activity and its downstream consequences. Note that SW620 cells with low expression of Sam69 exhibit interference between the ICG-001 and the pro-apoptotic action of butyrate. CBP acetylates Pygo2, enhancing nuclear localization and hence promoting basal Wnt activity and downstream effects of CBP-Wnt signaling. On the other hand, p300 acetylates Pygo2 to promote its localization to the cytoplasm, interfering with Pygo2-mediated Wnt activity and its physiological consequences. By affecting CBP activity, we hypothesize that Sam68 can also affect Pygo2 activity, linking the Sam68 and Pygo2 pathways through effects on CBP-Wnt vs. p300-Wnt activities and their downstream consequences.

The literature supports that (a) CBP and p300 play important roles in Wnt signaling and affect the ability of butyrate to modulate that signaling; (b) CBP- and p300-mediated activities can be modulated in colonic cells both genetically and pharmacologically; (c) p300 expression is correlated to CRC patient outcomes in a manner possibly related to fiber and butyrate; (d) deficient p300 expression correlates to butyrate resistance in several CRC cell lines; (e) Sam68 mediates the effects of ICG-001 in colonic cells by downregulating CBP-Wnt activity; and (f) the nucleocytoplasmic localization of Pygo2, controlled by CBP vs. p300 activity, affects Wnt signaling and colonic cell phenotypes. All these findings support the central hypothesis (Fig. 1): *Relative levels of Sam68 and Pygo2 affect CBP-Wnt and p300-Wnt activities, influencing response to ICG-001 and resistance to butyrate and other HDACis; thus, modulation of Sam68 and Pygo2 can enhance response to ICG-001 and HDACis, leading to more optimal preventive and therapeutic approaches against CRC.*

A key clinical finding underpinning the scientific premise of this hypothesis, and the need to investigate this hypothesis, is the relatively ineffective response of CRCs, and some other solid tumor cancers, to HDACis in clinical trials 50-52. One possibility, particularly for CRC, is that resistance to the HDACi activity of butyrate that develops during carcinogenesis causes the poor response of these cancers to HDACis in therapy. Investigating the hypothesis proposed here would evaluate how levels of factors that influence Wnt signaling can promote the development of resistance to HDACis, thus affecting treatment with these agents.

## Testing the hypothesis

### Cell lines

Colonic cell lines will be utilized that represent normal (CCD-841Con), microadenoma (earliest neoplastic stage, LT97), primary carcinoma (HT29, HCT-116, SW480) and metastatic (SW620) cells. In addition, a butyrate/HDACi-resistant CRC cell line (HCT-R) has been developed 2. HDACi-resistance is defined as the ability to proliferate at concentrations of HDACis that induce cell cycle arrest and apoptosis in parental cancer cells. In addition, resistant cells exhibit repressed activation of Wnt signaling when exposed to HDACis. The proposed experiments will also investigate a p300 knockout clone of HCT-116 and the matching Rescue line that has restored p300 expression.

### Section one: Determination of the effects of Sam68 on CBP- and p300-mediated Wnt activity, butyrate resistance, and colonic cell physiology

*Hypothesis: The levels of Sam68 in different cell types mediate the cell-type effects of ICG-001 and/or butyrate, and also mediate the effects of p300 depletion and consequent butyrate resistance.* Do levels of Sam68 in neoplastic colonic cells affect the physiological response to ICG-001/butyrate cotreatment and resistance to butyrate and other HDACis? Levels of Sam68 will be upregulated by expression vector or by the appropriate CRISPR activation plasmid. Sam68 expression will be repressed by the appropriate double nickase CRISPR system or by siRNA or shRNA. Cells will be treated by ICG-001 as previously described 44-47; HDACis used will be butyrate, a product of dietary fiber, as well as the clinically relevant HDACi LBH589, both administered as previously described 1,2,17,44-47, 53. Cells will be assayed for (a) complex formation between Sam68 and CBP, as well as between CBP and beta-catenin; (b) Wnt activity as measured by reporter assays; (c) proliferation; (d) apoptosis; and (e) clonal cell growth. With respect to measurements (b)-(e) the degree of resistance to butyrate and LBH589 will be determined, based on comparison to the degree to which these HDACis affect metrics (b)-(e) in HCT-116 cells (considered maximally sensitive) and HCT-R cells (considered maximally resistant). Nucleocytoplasmic fractionation will be utilized for protein isolation, to ascertain Sam68 localization. Cell lines assayed will be normal (CCD-841Con) colonic cells, microadenoma LT97 cells, primary carcinoma (HT29, HCT-116, SW480) and metastatic (SW620) cells. Included in these comparisons are the CSC-like (HT29) and non-CSC-like (SW480) CRC cells previously characterized with respect to Sam68 and response to ICG-001 42.

**Figure 2:**
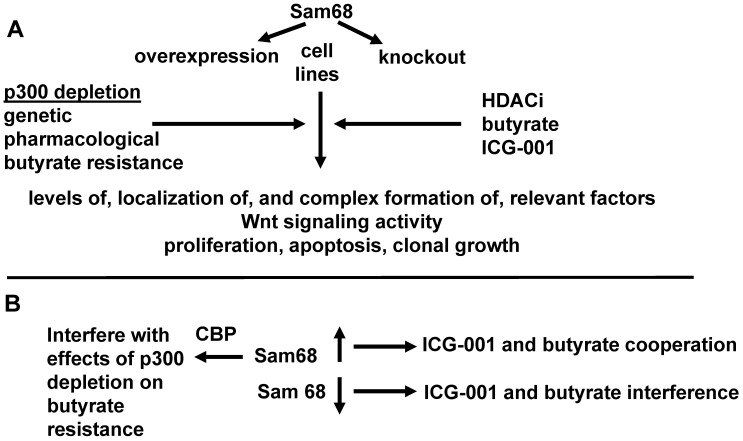
** Effects of Sam68 overexpression and repression on Wnt signaling and colonic cell physiology**. We present a more detailed hypothesis for section one, in the context of experimental approaches and expected results with respect to the hypothesis outlined in Fig. 1. **(A)** Sam68 will overexpressed or knocked out in cell lines treated with the HDACi butyrate and/or the CBP-Wnt inhibitor ICG-001. In some cell lines, p300 expression will be inhibited, by genetic means (knockout cells), pharmacological means (YH249), or in cells selected to be butyrate resistant than do not express p300 (HCT-R). We will evaluate the metrics listed. **(B)** We expect that overexpression of Sam68 will facilitate the interaction of ICG-001 and butyrate to induce apoptosis, while lowered levels of Sam68 will promote interference between those two agents with respect to induction of apoptosis. We also expect that higher levels of Sam68 would inhibit CBP-Wnt signaling and thus interfere with the ability of p300 depletion to promote butyrate resistance. These findings would constitute a confirmation of the hypothesis of section one.

Does Sam68 mediate the effects of altered p300 expression and/or activity on the physiology of neoplastic colonic cells, including development of resistance to butyrate and other HDACis? To explore the role of Sam68 in the effects of p300 depletion in CRC cells, particularly the phenomenon of resistance to butyrate, butyrate sensitive HCT-116 cell line (expresses p300) will be utilized, compared to butyrate resistant HCT-R cells that do not express p300, as well as the p300 KO HCT-116 line F5 that is partially butyrate resistant, and the p300 Rescue line in which p300 expression is restored to F5 cells, which, for the most part, restores butyrate sensitivity (Fig. 2) 44-47. p300-Wnt activity in HCT-116 cells will also be pharmacologically suppressed through the use of YH249, which blocks the association of p300 with beta-catenin 54 in analogous fashion to the action of ICG-001 against CBP-beta-catenin association. Under these conditions, Sam68 will be overexpressed or knocked out as described above. Cell assays will be performed as described above; in addition, we will also determine whether Sam68 associates with p300 or whether it has a highly specific association only with CBP.

### Section one: Expected results

It is expected that the levels of Sam68 will influence the effects of ICG-001 and butyrate cotreatment, in that higher levels of Sam68 expression will enhance the efficacy of ICG-001/butyrate to induce apoptosis, while lower levels of Sam68 will be associated with interference between ICG-001 and butyrate with respect to the induction of apoptosis. The depletion of p300 may affect, and be affected by, Sam68; however, Sam68 would mediate p300 depletion effects only if it can work in an ICG-001-independent fashion. It is expected that higher expression of Sam68 will interfere with the effects of p300 depletion with respect to butyrate resistance, since Sam68 would repress the CBP-Wnt activity presumably upregulated by p300 depletion. However, this assumes that the effects of Sam68 is specific to CBP and not p300 (which remains to be determined). Sam68 activity will be associated with its nuclear localization. Note that complete knockdown of Sam68 is likely not required to observe a phenotype, since lowered (but not absent) expression of Sam68 in SW480 cells was shown to be sufficient to alter response to ICG-001-like agents compared to HT29 cells that exhibit moderately higher levels of expression 42. The detailed hypothesis for section one, in the context of the experimental approach and expected results, is diagramed in Fig. 2.

### Section two: Identification of how altered CBP- and p300-mediated Wnt activity affects the localization and activity of Pygo2, whether these effects are mediated through the levels of Sam68, and we will determine the downstream effects of this altered cell signaling on colonic cell physiology

Hypothesis: The action of both ICG-001 and p300 depletion on Wnt signaling and cell physiology are mediated by Pygo2. ICG-001 will promote Pygo2 localization to the cytoplasm, while p300 depletion will promote Pygo2 localization to the nucleus. Thus, effects of ICG-001 and p300 depletion are affected by Sam68-controlled CBP-Wnt activity which in turn influences the localization and activity of Pygo2. Do levels, localization, and activity of Pygo2 in neoplastic colonic cells affect the physiological response to ICG-001/butyrate cotreatment, p300 depletion, and resistance to butyrate and other HDACis? Levels of Pygo2 will be upregulated by expression vector 43 or by CRISPR; Pygo2 expression will be knocked out by CRISPR. An expression vector for acetylation-deficient Pygo2 and one for acetylation-mimic Pygo2 43 will be utilized; the former being a repressive Pygo2 mutant vector and the later an activated Pygo2 mutant vector. Cells will be treated by ICG-001; HDACis used will be butyrate and LBH589. Cells will be assayed for (a) complex formation between Pygo2, CBP or p300, and beta-catenin; (b) Wnt activity as measured by reporter assays; (c) proliferation; (d) apoptosis; and (e) clonal cell growth. Resistance to HDACis will be evaluated as described in Section One. Nucleocytoplasmic protein isolation will be performed as described in Section One. Cell lines utilized will be the same as those used in both sections of Section One. In addition to the HCT-R and p300 knockout lines, p300-Wnt activity will also be repressed with YH249 as described above. Cell assay metrics will be conducted on untreated cells as well as cells treated with +/- ICG-001 +/- HDACis (butyrate or LBH589) as above. Exogenous expression of acetylation-deficient Pygo2 will be performed to confirm observations from Pygo2 knockdown; similar phenotypes are expected. The role of Pygo2 in observed effects will be confirmed through use of the acetylation-mimic Pygo2 expression vector, which is expected to potentiate observed effects of increased Pygo2 expression and activity, although whether the mimic potentiates both CBP- and p300-mediated effects remains to be empirically determined.

Does Pygo2 mediate the effects of Sam68 on Wnt activity and cell physiology, in neoplastic colonic cells? The hypothesis is that Pygo2 works downstream of Sam68 to mediate effects of Sam68 on Wnt signaling and colonic cell physiology, both in the untreated (basal) condition as well as with respect to treatment with ICG-001. First, the study would need to determine whether overexpression or knockdown of Sam68, as generated in Section One, alters the levels and nucleocytoplasmic localization of Pygo2.

In addition, to evaluate the possibility of feedback crosstalk between these two factors, the Pygo2-overexpressing and knockdown cells generated for Section 2 will be analyzed with respect to the levels and nucleocytoplasmic localization of Sam68 (+/- ICG-001), as well as CBP-Sam68 vs. CBP-beta-catenin association. To determine whether Pygo2 is a downstream effector of Sam68, the levels of both factors will be adjusted in tandem, in the following combinations: (a) overexpression of both Sam68 and Pygo2, (b) overexpression of Sam68 and knockdown of Pygo2, (c) knockdown of Sam68 and overexpression of Pygo2, and (d) knockdown of both Sam68 and Pygo2. This will be achieved by utilizing the Sam68 overexpression and knockdown cells generated in Section One, and, for each of these two lines, overexpressing and knocking down expression of Pygo2 as described above. Cell assay metrics (a)-(e) (+/- ICG-001 +/- HDACis) would then be performed as described above. These results could be confirmed by utilizing Pygo2 acetylation-deficient and Pygo2-mimic expression vectors.

### Section two: Expected results

**Figure 3:**
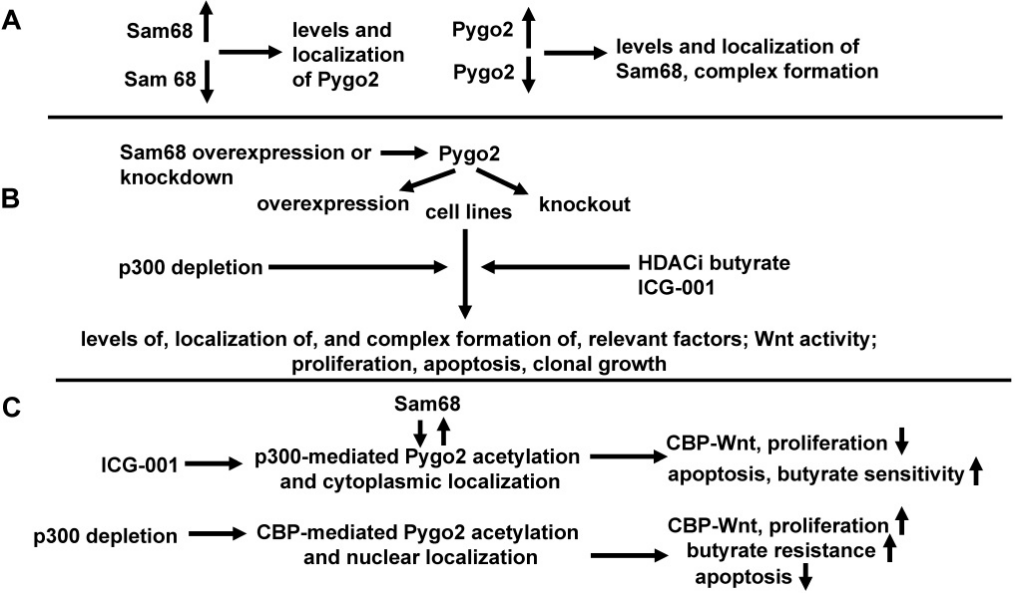
**Effects of Pygo2 overexpression and repression on Sam68 activity, Wnt signaling, and colonic cell physiology.** We present a more detailed hypothesis for section two, in the context of experimental approaches and expected results with respect to the hypothesis outlined in Fig. 1. **(A)** We will first ascertain if overexpression or repression of Sam68 affects the levels and localization of Pygo2, and whether overexpression or repression of Pygo2 affects the levels and localization of Sam68, as well as complex formation between Sam68 and CBP/beta-catenin. **(B)** Pygo2 will overexpressed or repressed in the presence or absence of overexpressed or repressed Sam68. Cell lines and treatments used, as well as the metrics evaluated, will be the same as described for section one (Fig. 2). In addition, findings generated with Pygo2 knockout will be conformed through the use of an expression vector for acetylation deficient Pygo2. **(C)** We expect that ICG-001, acting through Sam68 (itself influenced by Pygo2) will facilitate p300-mediated acetylation of Pygo2 and its cytoplasmic localization hence inhibiting CBP-Wnt activity and proliferation, while enhancing butyrate sensitivity and apoptosis, On the other hand, p300 depletion will enhance CBP-mediated Pygo2 acetylation and nuclear localization, enhancing CBP-Wnt activity, proliferation, and butyrate resistance, while inhibit apoptosis. These findings would constitute a confirmation of the hypothesis of section two.

ICG-001 decreases CBP-Wnt activity, while maintaining (or increasing) p300-Wnt activity 40. If the association of either CBP or p300 with beta-catenin is required for Pygo2 acetylation as has been suggested 43, one would expect ICG-001 to promote p300 acetylation of Pygo2 and Pygo2 cytoplasmic localization, resulting in suppressed CBP-Wnt activity (and consequent reduced expression of CBP-Wnt target genes), increased apoptosis and reduced cell proliferation; in addition, butyrate sensitivity would be enhanced. Depletion of p300 would, conversely, favor CBP acetylation of Pygo2 and Pygo2 nuclear localization, enhanced CBP-Wnt activity and consequent changes in CBP-Wnt-targeted gene expression; in addition, butyrate resistance would be induced. These expected results depend on requirement for CBP or p300 to be associated with beta-catenin for the altered acetylation and nucleocytoplasmic localization of Pygo2. Further, in conjunction with Section One, it is expected that Sam68 will affect Pygo2 acetylation and localization, and hence, its downstream effects. Thus, ICG-001 would, influenced by Sam68, deplete CBP-beta-catenin and favor Pygo2 acetylation by p300. Promoting a CBP pathway would lead to a more proliferative and butyrate resistant phenotype, while a p300 pathway would promote differentiation and butyrate sensitivity. Thus, effects of Sam68 will be dependent on Pygo2, so that the ability of Sam68 to influence Wnt signaling and cell physiology of neoplastic colonic cells will be maximal when both Sam68 and Pygo2 are overexpressed; knockdown of Pygo2 (or overexpression of the Pygo2 acetylation-deficient mutant) would repress Sam68-mediated changes on Wnt activity and cell physiology. These effects of Sam68 will be potentiated through overexpression of acetylation-mimic Pygo2. The detailed hypothesis for section two, in the context of experimental approach and expected results, is diagramed in Fig. 3.

## Conclusion

A major objective of investigating the hypothesis outlined here is to generate findings that will stimulate increased interest in the utility of HDACis in CRC prevention/therapy, and also increase understanding how the efficacy of HDACis may be enhanced through combinatorial therapy with ICG-001-like agents. The equivocal results of anti-CRC (and, in general anti-solid tumor) clinical trials involving HDACis 50-52 may be due to HDACi-resistance of these tumors; in the case of CRC, resistance may develop as a result of intra-colonic exposure to butyrate and consequent downregulation of p300-Wnt signaling. ICG-001-like agents have also been in clinical trial, and the findings of this study can enhance the efficacy not only of HDACi- or ICG-001-like-based treatments, but also combinatorial therapy using both classes of agents. Thus, the proposed experiments, and subsequent follow-up studies, may impact future treatment options by demonstrating how ICG-001-like agents and HDACis can be utilized more effectively against CRC**.** Further investigations along these lines may also provide a better understanding how Sam68 influences p300-mdiated alternative RNA splicing 55, which can affect gene expression and carcinogenesis.

## Abbreviations

HDACi: histone deacetylase inhibitor
CRC: colorectal cancer
CSC: cancer stem cell
Pygo2: Pygopus 2
